# Development of a Quality Evaluation Method for Allii Macrostemonis Bulbus Based on Solid-Phase Extraction–High-Performance Liquid Chromatography–Evaporative Light Scattering Detection Chromatographic Fingerprinting, Chemometrics, and Quantitative Analysis of Multi-Components via a Single-Marker Method

**DOI:** 10.3390/molecules29194600

**Published:** 2024-09-27

**Authors:** Jianfa Wu, Lulu Wang, Ying Cui, Chang Liu, Weixing Ding, Shen Ren, Rui Dong, Jing Zhang

**Affiliations:** 1 College of Chinese Medicinal Materials, Jilin Agricultural University, Changchun 130118, China; 20210971@mails.jlau.edu.cn (J.W.); 20210970@mails.jlau.edu.cn (Y.C.); 20210969@mails.jlau.edu.cn (C.L.); 20221576@mails.jlau.edu.cn (W.D.); 20221619@mails.jlau.edu.cn (S.R.); 2 School of Medicine, Changchun Sci-Tech University, Changchun 130600, China; 20191188@mails.jlau.edu.cn; 3 Jilin Provincial International Joint Research Center for the Development and Utilization of Authentic Medicinal Materials, Changchun 130600, China

**Keywords:** Allii Macrostemonis Bulbus, fingerprint, chemometrics, quantitative analysis of multi-components by single-marker

## Abstract

As a traditional Chinese medicine (TCM), Allii Macrostemonis Bulbus (AMB) is a key herb for the treatment of thoracic paralytic cardiac pain, but its quality evaluation method has not yet been fully clarified. In this study, chromatographic fingerprints of AMB were developed using solid-phase extraction–high-performance liquid chromatography–evaporative light scattering detection (SPE-HPLC-ELSD) to evaluate the quality of AMB from various origins and processing methods. This was achieved by employing chemical pattern recognition techniques and verifying the feasibility and applicability of the quality evaluation of AMB through the quantitative analysis of multi-components via a single-marker (QAMS) method. Through the analysis of the fingerprints of 18 batches of AMB, 30 common peaks were screened, and 6 components (adenosine, syringin, macrostemonoside T, macrostemonoside A, macrostemonoside U, and macrostemonoside V) were identified. Moreover, three differential markers (macrostemonoside A, macrostemonoside T, and macrostemonoside U) were screened out using chemometrics techniques, including principal component analysis (PCA), hierarchical cluster analysis (HCA), and orthogonal partial least squares discriminant analysis (OPLS-DA). Subsequently, a QAMS method was established for macrostemonoside T and macrostemonoside U using macrostemonoside A as an internal reference. The results demonstrate the method’s accuracy, reproducibility, and stability, rendering it suitable for the quality evaluation of AMB. This study provides a theoretical basis for drug quality control and the discovery of quality markers for AMB.

## 1. Introduction

As is widely acknowledged, traditional Chinese medicines (TCMs) have garnered increasing global attention due to their characteristics of being multi-component, multi-target, and low in toxicity [1]. Allii Macrostemonis Bulbus (AMB), belonging to the genus *Allium* in the Liliaceae family, stands as a traditional medicinal and culinary plant in China, often referred to as the “*Ganoderma lucidum* among vegetables” [2]. AMB has historically been recognized as a crucial herb for conditions such as “chest stuffiness and pains” and has been used for millennia to address cardiovascular diseases, diarrhea, abdominal pain, and asthma [3]. Modern pharmacological studies have shown that AMB possesses a variety of pharmacological activities such as antimyocardial injury [4], antiplatelet aggregation [5], antioxidant [6], and anticancer properties [7]. Concurrently, AMB has been utilized as a culinary vegetable. According to yaozhi.com (accessed on 20 June 2024) [8], AMB is currently integrated into four types of health food, eighteen TCM formulae, and sixty-four TCM prescriptions, underscoring its substantial developmental potential. Studies have identified chemical components within AMB, including steroidal saponins, volatile oils, flavonoids, phenylpropanoids, alkaloids, and polysaccharides, exhibiting pharmacological activities such as cardiovascular protection, antioxidation, antitumor properties, and antidepressant effects [9]. However, the quality of AMB herbs is susceptible to various factors like origin, climate, and processing methods [9]. In addition, AMB has two botanical sources, both in the genus *Allium*, in the family Liliaceae, *Allium macrostemon* Bunge and *Allium chinense* G. Don, respectively, which closely resemble each other in appearance post-processing, posing challenges in distinguishing their sources.

Despite the limited components isolated from AMB, the 2020 edition of the *Chinese Pharmacopoeia* relies solely on microscopic identification and thin-layer chromatography, lacking specifications regarding the quality markers of AMB [10]. Consequently, there is a pressing need to devise a new method for comprehensive quality control of AMB. Fingerprinting, acknowledged by authorities such as the World Health Organization (WHO), the U.S. Food and Drug Administration (FDA), and the State Food and Drug Administration of China (SFDA), among others, stands as an alternative means to authenticate and ensure the consistency of TCMs quality [11]. Chromatographic fingerprinting of TCMs serves as an exhaustive and measurable phytochemical identification tool, characterized by its fundamental traits of “completeness” and “ambiguity” [12]. However, parameters concerning the quality of TCMs derived from chromatographic fingerprints of TCMs present an extensive dataset. Such data can only be suitably and systematically processed through chemometric calculations [13]. Multimodal analysis methods developed in chemometrics enable chromatographic fingerprinting to be used as a technique for identifying varieties and evaluating the quality of Chinese herbal medicines, Chinese herbal medicinal drinks, Chinese herbal formulae, and proprietary Chinese medicines. The complexity of the components in Chinese medicines makes it difficult to assess their quality through single-component or index evaluations. In a previous study, the quality of AMB was evaluated via ultra-high performance liquid chromatography quadrupole time-of-flight tandem mass spectrometry (UPLC-TOF-MS), but the traditional external standard method (ESM) presents challenges such as difficulty in standard separation, instability in nature, and high expense [14]. As a result, the quantitative analysis of multi-components by single-marker (QAMS) method has emerged. This method achieves simultaneous determination of multiple component contents by calculating the relative correction factors (RCFs) between each chemical component of TCMs and a single reference standard. It offers advantages such as lower cost and shorter cycle times [15,16,17].

The 2020 edition of the *Chinese Pharmacopoeia* has incorporated QAMS assays for 15 TCMs, including Coptidis Rhizoma, Zingiberis Rhizoma Recens, and Salviae Miltiorrhizae Radix et Rhizoma [10]. Additionally, the QAMS method has garnered continuous attention in the international natural medicine quality control research field. The 38th edition of the *United States Pharmacopoeia* includes the QAMS method in the quality standards for a total of 90 botanical drugs, while the 8th edition of the *European Pharmacopoeia* applies the QAMS method to 23 botanical drug standards [16]. Due to the poor UV absorption of some saponins in AMB, the high-performance liquid chromatography–tandem evaporative light scattering detector (HPLC-ELSD), which can detect components without UV absorption, has found widespread use in the quality evaluation of Chinese herbal medicines, Chinese herbal formulas, and proprietary Chinese medicines [18,19,20]. Additionally, solid-phase extraction (SPE), which exploits differences in the adsorption capacity of components in different mobile phases, can effectively separate target components from interfering components, enhancing the detection rate of the target components. SPE has also been widely employed as a sample pretreatment technique [21,22].

In this study, we employed SPE-HPLC-ELSD to establish the fingerprints of AMB and utilized the QAMS method to determine the multicomponent content of the shared components in AMB. Additionally, we integrated chemometric methods such as similarity analysis (SA), principal component analysis (PCA), cluster analysis (HCA), and orthogonal partial least squares discriminant analysis (OPLS-DA) to conduct quality evaluation. To the best of our knowledge, this method has not been previously explored for assessing the quality of AMB. It proves to be effective, convenient, and practical, offering the potential to provide updated information on AMB’s quality control. Moreover, it provides a theoretical foundation for enhancing the quality evaluation system of AMB and identifying quality markers.

## 2. Results and Discussion

### 2.1. Examination of Chromatographic Conditions

In this study, the HPLC system was optimized for different mobile phases (methanol–water, acetonitrile–water, methanol–0.05% formic acid in water, and acetonitrile–0.05% formic acid in water). Columns [Hypersil ODS (250 mm × 4.6 mm, 5 µm), Agilent TC-C_18_ (250 mm × 4.6 mm, 5 µm) and COSMOSIL 5C_18_-MS-II (250 mm × 4.6 mm, 5 µm)], flow rates (0.6, 0.8, and 1 mL·min^−1^), column temperatures (25 °C, 30 °C, and 35 °C), and injection volumes (10, 15, and 20 μL) were examined and optimized. The drift tube temperatures (60 °C, 70 °C, 80 °C, 90 °C, and 100 °C), gas flow rates (1.5, 2.0, 2.5, and 3.0 L·min^−1^), and gain values (1, 2, and 3) were examined and optimized in the ELSD system. Since the components in AMB are diverse and exhibit different polarities, it is difficult to separate them by isocratic elution. Therefore, this study employed gradient elution and evaluated the suitability of chromatographic conditions by examining the number of peaks, peak shapes, and separations as indicators. It was found that the effects of column and column temperature on the chromatographic peaks were minimal, while the effects of other conditions on the chromatographic peaks were large. Finally, the optimal chromatographic conditions were determined to be an Agilent TC-C_18_ (250 mm × 4.6 mm, 5 µm) column with acetonitrile–0.05% formic acid in water as the mobile phase at a flow rate of 0.8 mL·min^−1^, a column temperature of 35 °C, a feed volume of 20 μL, a drift tube temperature of 90 °C, a gas flow rate of 2.0 L·min^−1^, and a gain value of 3.

### 2.2. Establishment of Fingerprints and Similarity Analysis (SA)

**Figure 1:**
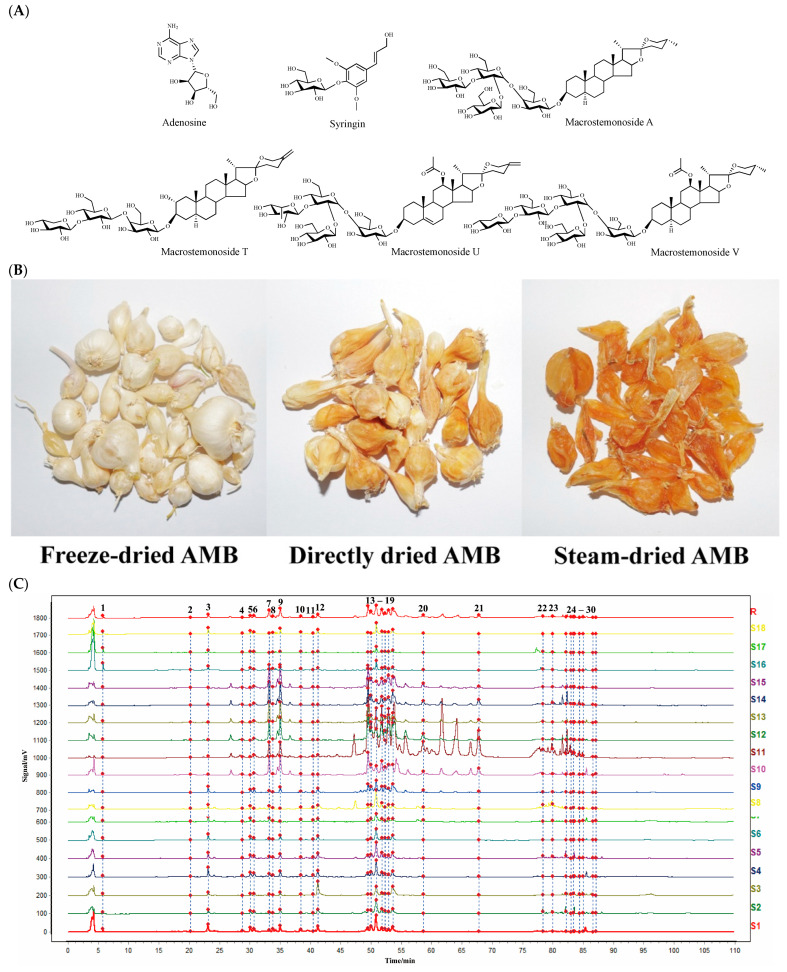

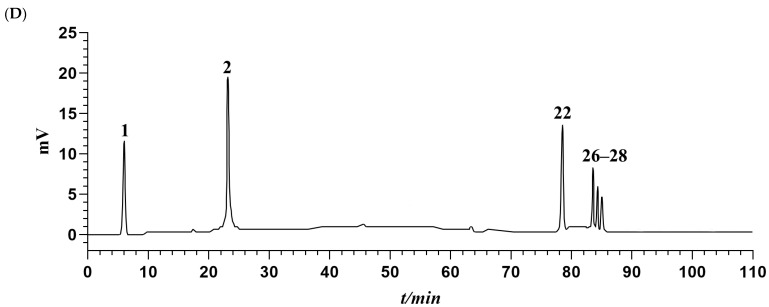
(**A**) Structures of the six identified components. (**B**) Appearance properties of three AMB processed products. (**C**) Fingerprints of AMB for different origins and processing methods (18 batches). (**D**) Mixed standard HPLC-ELSD profiles of six components (1: adenosine; 2: syringin; 22: macrostemonoside T; 26: macrostemonoside A; 27: macrostemonoside U; 28: macrostemonoside V).

The chromatographic data from S1 to S18 were input into the *Chinese Medicine Chromatographic Fingerprint Similarity Evaluation System* (2012 version). Using S1 as the reference spectrum and a time window width of 0.1 min, standardized fingerprints for AMB from different origins and processing methods were generated using the average method (Figure 1C). Across the 18 samples, 30 common peaks were identified. Among these, peaks 1, 2, 22, 26, 27, and 28 were specifically identified as adenosine, syringin, macrostemonoside T, macrostemonoside A, macrostemonoside U, and macrostemonoside V, respectively (Figure 1D). Moreover, the similarity between the fingerprint profiles of S1 to S18 and the control profile (R) was calculated using multipoint correction and mark peak matching, detailed in Table 1. For most samples, the similarity to the control profile exceeded 0.9. It is noteworthy that the similarity values for S10 to S15 were below 0.8. This discrepancy is attributed to the geographic and climatic differences between the southern and northern provinces of China, from which these samples were obtained. Visual inspection of the fingerprints of AMB from these regions revealed more pronounced peaks in those from the southern provinces. This distinction was consistent with the higher presence or content of several peaks in the fingerprints of AMB from the southern provinces compared to those from the northern provinces.

### 2.3. Examination of HPLC Fingerprinting and Quantification Methods

#### 2.3.1. Linear Range Examination

We assessed the linear range by preparing five concentrations and conducting linear regression using the least squares method. Adenosine, syringin, macrostemonoside T, macrostemonoside A, macrostemonoside U, and macrostemonoside V were chosen as indicators for this assessment. Linear regression equations were derived by plotting the concentration (*x*) against the peak area (*y*) of different standards. The determination coefficients (R^2^) for all the standards across varying concentrations exceeded 0.996, demonstrating a robust linear correlation between the concentrations of these six components and their respective peak areas within the tested concentration range (Table 2).

#### 2.3.2. Precision, Stability, and Repeatability Examination

The precision assessment revealed RSD values ranging between 0.24% and 0.80% for the six components, indicating excellent method precision. Stability testing further indicated RSD values between 0.31% and 0.73%, affirming the stability and reliability of the AMB sample solution within a 24 h timeframe. Repeatability analysis demonstrated RSD values below 2.0% for all six components, showcasing strong precision and satisfactory reproducibility of the sample assay. Detailed results for these assessments are provided in Table 2.

#### 2.3.3. Examination of LOD, LOQ, and Spiking Recoveries

The LOD and LOQ values for the six components are detailed in Table 2. Moreover, the average recoveries for adenosine, syringin, macrostemonoside T, macrostemonoside A, macrostemonoside U, and macrostemonoside V were 103.31%, 102.23%, 102.56%, 101.63%, 98.45%, and 98.21%, respectively, with RSD values below 2.0% for each. These findings affirm the accuracy and reliability of the method (Table 2).

However, relying solely on fingerprint analysis did not provide a comprehensive and objective examination of how processing methods influence variations in AMB composition. Therefore, this study employed chemometric techniques to analyze the chromatographic data comprehensively.

### 2.4. Principal Component Analysis (PCA)

**Figure 2:**
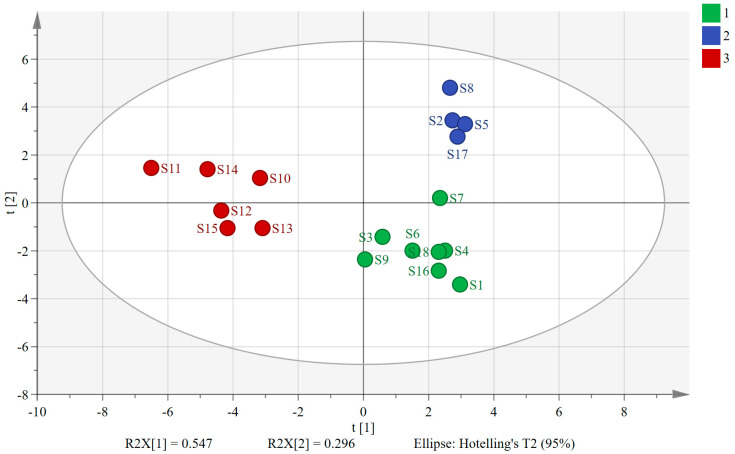
Plot of PCA scores for 18 batches of AMB of different origins and processing methods (1: AMB treated via direct drying and post-steaming drying in northern production areas; 2: AMB treated via freeze-drying in northern production areas; 3: AMB in southern production areas).

PCA, a dimensionality reduction analysis model, condenses multiple metrics into a few composite metrics while exploring their correlations [23,24,25]. By applying *SIMCA 14.1* software, we used the peak areas of the 30 shared peaks as variables and extracted two principal components. The variance contributions of these two principal components were 54.7% and 29.6%, respectively, and the cumulative variance contribution (R^2^) was 84.3%, indicating that the two extracted principal components were able to characterize the information of the herbs in a relatively comprehensive manner. The model exhibited a cumulative predictive rate (Q^2^) of 0.696, demonstrating strong fitting and predictive abilities. The PCA scatter plots in Figure 2 visually depict similarities and differences between samples, with sample relationships determined by the distances between scatter points. Notably, the 18 sample batches formed three distinct clusters, with S10–S15 (sourced from southern provinces) grouped on the left. Conversely, S1–S9 and S16–S18 (from northern provinces) were categorized on the right. Interestingly, within the clusters on the right side, S2, S5, S8, and S17 (processed via freeze-drying) formed a distinct cluster, while the remaining samples clustered separately, being primarily composed of AMB processed through direct drying or post-steaming drying methods. These findings underscore the significant influence of both geographic origin and processing method on the overall quality of AMB. Notably, the geographic environment exerted a stronger impact than the processing method.

### 2.5. Hierarchical Cluster Analysis (HCA)

**Figure 3:**
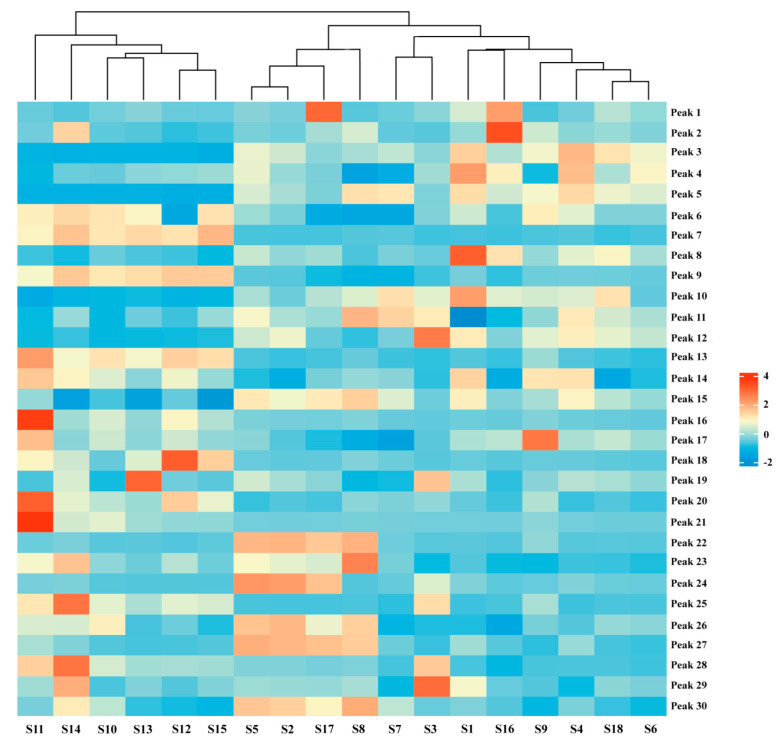
Hierarchical clustering heat map of 18 batches of AMB of different origins and processing methods.

HCA, an unsupervised clustering method, constructs nested trees of clusters by assessing similarities based on squared Euclidean distances [26,27]. Using *Origin Pro* software (https://www.originlab.com/) and the peak areas of the 30 shared peaks as variables, we clustered AMB from various origins and processing methods. The resulting heat maps, which depict the impact of variables on clustering, are presented in Figure 3. The 18 sample batches were broadly divided into two clusters: AMB from southern Chinese provinces (S10–S15) and AMB from northern provinces (S1–S9 and S16–S18). Notably, within the northern provinces, a further subdivision emerged into two distinct clusters: AMB processed via freeze-drying (S2, S5, S8, and S17) and those processed through direct drying or post-steaming drying methods (S1, S3, S4, S6, S7, S9, S16, and S18). These findings align with the outcomes of the PCA analysis. The HCA results highlighted that differences in AMB components were significantly influenced by both origin and processing methods, potentially related to regional climate and soil variations between northern and southern China. Furthermore, distinctions between AMB batches may also correlate with the application of heat during processing.

### 2.6. Orthogonal Partial Least Squares-Discriminant Analysis (OPLS-DA)

We conducted a supervised OPLS-DA analysis following PCA, aiming to further explore intergroup differences among AMB of varying origins and processing methods, focusing on identifying markers contributing significantly to these differences [28]. Using the peak areas of 30 common peaks from 18 AMB samples as variables in *SIMCA 14.1* software, the OPLS-DA model had a fit index of 0.753 for the independent variables ($R_{x}^{2}$), a fit index of 0.992 for the dependent variables ($R_{y}^{2}$), and a predictive index (Q^2^) of 0.993. These indices, with both R^2^ and Q^2^ surpassing 0.5, validated the model’s reliability [29].

**Figure 4:**
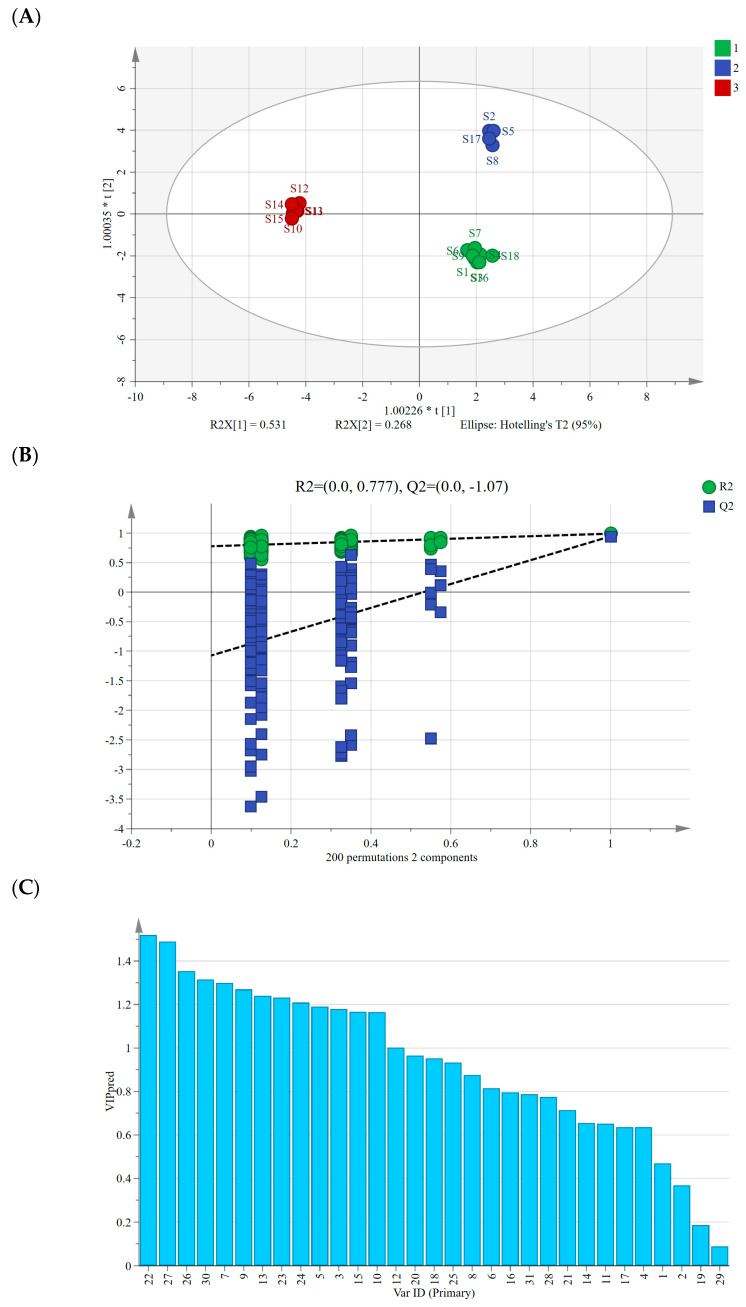
(**A**) Plot of OPLS-DA scores for AMB of different origins and processing methods. (**B**) Model cross-validation plot. (**C**) VIP values for 30 shared peaks.

Figure 4A displays the OPLS-DA scores’ scatter plot, revealing the differentiation of AMB based on their origin, distinguishing between the southern and northern production regions. Similar to PCA and HCA results, the AMB from northern production regions further segregated into freeze-dried and directly dried or post-steaming dried categories along the horizontal axis. After the 200 permutation tests depicted in Figure 4B, higher R^2^ and Q^2^ values on the right side and the Q^2^ regression line intercepting below 0 (−1.07) affirmed the model’s validation without overfitting, ensuring its utility in identifying differential markers [30]. Moreover, we computed the variable importance in projection (VIP) values, considering VIP > 1 as a criterion for differential marker screening among the 18 AMB batches [31]. A total of 13 differential markers were identified (Figure 4C), including peaks 22, 27, 26, 30, 7, 9, 13, 23, 24, 5, 3, 10, and 12, with VIP values of 1.52, 1.49, 1.35, 1.32, 1.30, 1.27, 1.24, 1.23, 1.21, 1.19, 1.18, 1.17, 1.16, and 1.00. Among them, peaks 22, 27, and 26 were identified as macrostemonoside T, macrostemonoside U, and macrostemonoside A, respectively, which can be used as differential markers affecting the quality of AMB.

### 2.7. Establishment of the Evaluation Model of the Quantitative Analysis of Multi-Components by Single-Marker (QAMS) Method

Since research on macrostemonoside A began earlier [32,33,34] and the isolation technique was relatively mature, macrostemonoside A was indeed the most suitable internal reference of macrostemonoside T and macrostemonoside U. The results showed that macrostemonoside A was the best choice for the determination of macrostemonoside T and macrostemonoside U. The results can be summarized as follows.

#### 2.7.1. Calculation of Relative Correction Factors (RCFs)

The peak areas of the internal reference and the components to be tested were determined by ESM, and the RCFs of the components to be tested and the internal reference were calculated via multiple concentrations according to the formula RCF = A_s_·A_i_/(A_i_·C_s_), where A_s_ and A_i_ are the peak areas of the internal reference and the components to be tested, and C_s_ and C_i_ are the mass concentrations of the internal reference and the component control to be tested, respectively. The RCFs for macrostemonoside T and macrostemonoside U with respect to macrostemonoside A were 2.56 and 1.74, with RSD values of 1.35% and 1.58%, respectively.

#### 2.7.2. Examination of Durability

Different columns [Hypersil ODS (250 mm × 4.6 mm, 5 µm), Agilent TC-C_18_ (250 mm × 4.6 mm, 5 µm), and COSMOSIL 5C_18_-MS-II (250 mm × 4.6 mm, 5 µm)], flow rates (0.6, 0.8, and 1 mL·min^−1^), column temperatures (25 °C, 30 °C, and 35 °C), and injection volumes (10, 15, and 20 μL) were selected. The results showed that the RSD of the RCFs of macrostemonoside T and macrostemonoside U with macrostemonoside A were less than 2.0% (Table 3), indicating that the effects of different columns, flow rates, column temperatures, and injection volumes on the determination of macrostemonoside T and macrostemonoside U in the AMB were not significant.

#### 2.7.3. Chromatographic Peak Localization

The relative retention time (R_t_ = t_i_/t_s_) was used as a criterion for the localization of the chromatographic peaks. Here, R_t_ represents the relative retention time, t_i_ represents the retention time of the component being tested, and t_s_ represents the retention time of the internal reference material. A total of 20 μL of mixed standard 2 were injected, respectively, to examine the effects of the above three chromatographic columns on the R_t_. The results showed that the R_t_ values for macrostemonoside T and macrostemonoside U with respect to macrostemonoside A were 0.9394 and 1.0098, respectively. The R_t_ fluctuation of each component was small, with RSD values of 0.18% and 0.15%, respectively, indicating no significant difference. This suggests that different columns can be utilized to analyze the effect of Rt for localizing macrostemonoside T and macrostemonoside U in AMB.

### 2.8. Content Determination

Eighteen batches of AMB samples (S1–S18) were prepared according to the method described in Section 3.3. Three samples from each batch were prepared in parallel, measured using the chromatographic conditions outlined in Section 3.4, and their peak areas were recorded. These peak areas were then calculated using both QAMS and ESM. The relative deviation (RD) was calculated to verify the accuracy of the RCFs. The results are shown in Table 4, with RD values for the comparison of both methods falling between ± 5.0%, indicating no significant difference between the ESM and QAMS content determination results. Interestingly, the content of the three components in the directly dried processed AMB was significantly higher than in the post-steaming dried and lyophilization processed AMB across all 18 batches, providing insight into AMB’s processing methods.

These results indicate that the QAMS method established, with macrostemonoside A as an internal reference, is efficient, reliable, and accurate, and can be used for the quality evaluation of AMB.

## 3. Materials and Methods

### 3.1. Chemicals and Materials

Adenosine (Item No. A108809) and syringin (Item No. S101544) were purchased from Aladdin Biochemical Science and Technology Co., Ltd. (Shanghai, China). In a previous study [6,35], we obtained macrostemonoside T, macrostemonoside A, macrostemonoside U, and macrostemonoside V. The purity of all components was ≥98% as determined by area normalization, and the structures are shown in Figure 1A. Acetonitrile (HPLC grade, ≥99.99%) was purchased from Thermo Fisher Scientific (Waltham, MA, USA), formic acid (HPLC grade, ≥95%) was purchased from Sigma-Aldrich (St. Louis, MO, USA), methanol (HPLC grade, ≥99.5%) was purchased from Tianjin Tiantai Chemicals Co., Ltd. (Tianjin, China), and purified water was obtained from Wahaha Group Co., Ltd. (Hangzhou, China). SEP-PAK C_18_ solid-phase extraction columns (SPEC) were purchased from Waters Corporation (Milford, MA, USA). Different batches of AMB were obtained from different provinces in China, and these samples were identified as *Allium macrostemon* Bunge, a species of AMB. The species of the samples were identified by Prof. Jing Zhang of Jilin Agricultural University, and the samples were kept in the sample bank of the College of Traditional Chinese Medicinal Materials of Jilin Agricultural University (No. 20210971).

### 3.2. Processing of Allii Macrostemonis Bulbus

Fresh AMB from different origins in China were processed into various products using the following methods: (1) AMB was pre-cooled at −20 °C and then placed in a LYO QUEST-55 vacuum freeze-dryer (Telstar Co., Ltd., Terrassa, Spain), where it was lyophilized to obtain freeze-dried AMB; (2) AMB was placed in a DHG-9070A electrically heated constant-temperature blower drying oven (Shanghai Shenxian Thermostat Equipment Factory, Shanghai, China), and the moisture within the herbs was removed at 50 °C to obtain directly dried AMB; (3) AMB was put into a steamer and steamed through and the surface water was wiped off. The remaining water in the herb was then removed using an electrically heated blast dryer at 50 °C to obtain steam-dried AMB. The different processed products of AMB in each production area are detailed in Table 1 (Numbered S1–S18). The appearance properties of the three processed products are shown in Figure 1B.

### 3.3. Preparation and Purification of Standards and Test Samples

Standard 1 [adenosine (2.71 mg·mL^−1^), syringin (1.36 mg·mL^−1^), macrostemonoside T (0.92 mg·mL^−1^), macrostemonoside A (0.23 mg·mL^−1^), macrostemonoside U (0.13 mg·mL^−1^), and macrostemonoside V (0.12 mg·mL^−1^)]; and standard 2 [macrostemonoside T (0.23 mg·mL^−1^), macrostemonoside A (0.21 mg·mL^−1^), and macrostemonoside U (0.23 mg·mL^−1^)] were weighed precisely, dissolved in 80% methanol–water solution, and then passed through 0.22 μm microporous filter membrane to obtain two mixed standard solutions.

AMB from different origins and processing methods were crushed and sieved through a 40-mesh sieve. A total of 2.00 g of AMB powder was weighed precisely, and 80% methanol–water was added in the ratio of 1:30 (m: V, g·mL^−1^). Each group was weighed and extracted via ultrasonic extraction at 50 °C for 60 min at 100 W. After cooling, the corresponding solvents were added to make up for the loss in weight. The extraction process was repeated three times, and the filtrates were combined to recover the solvents under reduced pressure, which gave the AMB extract. Since the AMB extracts contained a large number of impurities, which affected the subsequent chromatographic analysis, SPEC was used to purify each sample. Each AMB extract was dissolved in water, added to the activated SPEC, and washed with 10% methanol–water for 5 column volumes to remove impurities. Subsequently, the analyte was then eluted with 5 column volumes of methanol. The solvent was recovered under reduced pressure, and 2 mL of 80% methanol–water solution of the sample was added to the sample, which was passed through 0.22 μm microporous filtration membrane to obtain the AMB test material.

### 3.4. Chromatographic Conditions

Based on our previous research [35], the analysis was performed using an Acchrom S6000 high-performance liquid chromatograph (HPLC) (Acchrom Tech Technology Co., Ltd., Beijing, China), coupled with an ELSD-UM 5800 Plus detector (Unimicro Technologies Co., Ltd., Shanghai, China). The chromatographic column utilized was an Agilent TC-C18 (250 mm × 4.6 mm, 5 µm) maintained at 35 °C. The injection volume was set at 20 μL, with a flow set at 0.8 mL·min^−1^. Additionally, the evaporation temperature was set to 90 °C, the gas flow rate of nitrogen to 2.0 L·min^−1^, and the gain value was adjusted to 3. Mobile phase A consisted of acetonitrile, while mobile phase B comprised a 0.05% formic acid-water solution. The elution was carried out by gradient elution following the procedure in Table 5.

### 3.5. Methodological Examination

#### 3.5.1. Examination of HPLC Fingerprinting Methods

The precision test was performed by injecting the mixed standard 1 solution into the sample six times consecutively and calculating the relative standard deviation (RSD) value for each standard. Sample S1 was injected six times consecutively and the RSD value of each standard was calculated to assess the repeatability of the injection process. Sample S1 was left for 0, 2, 4, 8, 16, and 24 h before injection, and the RSD value of each standard was calculated to assess the stability of the sample over time before injection.

#### 3.5.2. Examination of HPLC Quantification Methods

The limit of detection (LOD) and limit of quantification (LOQ) were 3 and 10 times the signal-to-noise ratio, respectively. Calibration curves were plotted by injecting standards at various concentrations (*x*) and measuring the corresponding peak areas (*y*). These curves were then used to determine the content of six standards (adenosine, syringin, macrostemonoside T, macrostemonoside A, macrostemonoside U, and macrostemonoside V) in different batches of AMB. A sample of AMB powder (S1) with a known content was weighed. Then, a mixed control solution containing equal amounts of each component from the herb powder was prepared. Six sample solutions were prepared as described and injected as described above for determination. Subsequently, the spiked recoveries and RSD values of each standard were calculated.

### 3.6. Data Analysis and Chemometric Methods

The chromatographic fingerprints of AMB were established by the *Chinese Medicine Chromatographic Fingerprints Similarity Evaluation System* software (version 2012), the control chromatographic fingerprints were created, and the SA data and the peak areas of the common peaks were obtained for different batches of AMB. The obtained data were analyzed via chemometrics such as PCA and OPLS-DA using *SIMCA-P* software (version 14.1). The data were analyzed via a clustering heat map using *Origin Pro* software (version 2022).

## 4. Conclusions

In this study, we established SPE-HPLC-ELSD fingerprints for 18 batches of AMB sourced from various origins and processed using different methods. We identified 30 common peaks, six of which were successfully characterized. By employing a combination of fingerprint similarity evaluation and chemometric analyses, we categorized these 18 samples into three clusters. This classification revealed that the intrinsic quality of AMB is influenced by both origin and processing methods, with the geographic origin exerting a stronger influence than the processing methods. Furthermore, through OPLS-DA analysis, three differential markers (macrostemonoside A, macrostemonoside T, and macrostemonoside U) were identified in AMB. Macrostemonoside A served as an internal standard for analyzing macrostemonoside T and macrostemonoside U. Comparative analysis of the QAMS method and ESM showed no significant differences. Despite these research results, some limitations remain. Due to AMB’s complex chemical composition, identifying all components can be challenging, and fully characterizing AMB, especially distinguishing major components from impurities, remains difficult. While SPE-HPLC-ELSD and QAMS are effective for assessing AMB quality, they may have limitations in detecting certain components or providing detailed structural information. Therefore, future studies could explore complementary analytical techniques (e.g., liquid chromatography–mass spectrometry) to overcome these limitations and provide a more comprehensive understanding of the chemical composition and quality of AMB. In conclusion, this study presents a viable method for evaluating and categorizing the quality of AMB derived from diverse origins and processed using various methods. Additionally, our findings provide new insights into discovering quality markers in AMB.

## Figures and Tables

**Table 1:** The similarity of AMB from different origins and processing methods.

AMB	Origins	Processing Methods	Similarity
S1	Changchun, Jilin	Post-steaming dried	0.942
S2	Changchun, Jilin	Directly dried	0.926
S3	Changchun, Jilin	Freeze dried	0.924
S4	Benxi, Liaoning	Post-steaming dried	0.993
S5	Benxi, Liaoning	Directly dried	0.937
S6	Benxi, Liaoning	Freeze dried	0.971
S7	Luoyang, Henan	Post-steaming dried	0.921
S8	Luoyang, Henan	Directly dried	0.911
S9	Luoyang, Henan	Freeze dried	0.910
S10	Zigong, Sichuan	Post-steaming dried	0.745
S11	Zigong, Sichuan	Directly dried	0.753
S12	Zigong, Sichuan	Freeze dried	0.742
S13	Bijie, Guizhou	Post-steaming dried	0.720
S14	Bijie, Guizhou	Directly dried	0.685
S15	Bijie, Guizhou	Freeze dried	0.626
S16	Changchun, Jilin	Post-steaming dried	0.940
S17	Changchun, Jilin	Directly dried	0.915
S18	Changchun, Jilin	Freeze dried	0.959

**Table 2:** The linear range, LOD, LOQ, RSD values (%) for precision, stability, reproducibility, and spiked recoveries of the six standards (*n* = 6).

Components	Standard Curves	Content Ranges (μg·mL^−1^)	R^2^	LOD (μg·mL^−1^)	LOQ (μg·mL^−1^)	Precision	Stability	Repeatability	Spiked Recovery
Adenosine	*y* = 2463.0*x* − 127.53	6.62–109.11	0.9988	1.43	4.76	0.24	0.51	1.18	1.74
Syringin	*y* = 1254.7*x* − 78.93	14.24–96.31	0.9974	3.53	11.78	0.80	0.64	1.00	1.14
Macrostemonoside T	*y* = 861.0*x* − 69.574	25.83–81.35	0.9968	5.71	19.05	0.41	0.31	1.02	0.46
Macrostemonoside A	*y* = 2223.2*x* − 117.7	21.42–72.31	0.9970	5.67	18.90	0.34	0.54	1.00	0.62
Macrostemonoside U	*y* = 1308.1*x* − 71.569	24.37–86.44	0.9965	5.76	19.19	0.53	0.59	0.69	0.91
Macrostemonoside V	*y* = 1638.4*x* − 80.186	31.08–89.37	0.9983	8.16	27.20	0.68	0.73	0.84	1.07

**Table 3:** RCFs of components to be measured on different chromatographic columns, flow rates, column temperatures, and injection volumes (*n* = 3).

Durability Examination Indicators		Macrostemonoside T	Macrostemonoside U
Chromatographic columns	Hypersil ODS	2.55	1.73
	Agilent TC-C_18_	2.56	1.74
	COSMOSIL 5C_18_-MS-II	2.57	1.74
	RSD, %	0.47	0.37
Flow rates (mL·min^−1^)	0.6	2.55	1.74
	0.8	2.58	1.76
	1.0	2.56	1.72
	RSD, %	0.43	1.21
Column temperatures (°C)	25	2.56	1.73
	30	2.56	1.74
	35	2.57	1.73
	RSD, %	0.2	0.33
Injection volumes (μL)	10	2.57	1.78
	15	2.53	1.73
	20	2.58	1.72
	RSD, %	1.13	1.79

**Table 4:** QAMS and ESM determination of three components in 18 batches of AMB (*n* = 3).

No.	Macrostemonoside A	Macrostemonoside T			Macrostemonoside U		
	ESM, μg·g^−1^	ESM, μg·g^−1^	QAMS, μg·g^−1^	RD, %	ESM, μg·g^−1^	QAMS, μg·g^−1^	RD, %
S1	66.33	102.20	101.73	−0.46	77.98	77.15	−1.06
S2	168.46	346.97	345.51	−0.42	98.59	97.94	−0.66
S3	95.77	101.75	101.78	0.03	198.86	197.75	−0.56
S4	89.38	101.75	101.31	−0.43	84.27	84.07	−0.24
S5	180.09	344.05	344.87	0.24	98.09	97.90	−0.19
S6	96.92	102.25	102.10	−0.15	191.63	191.15	−0.25
S7	75.26	108.41	108.12	−0.27	92.24	91.92	−0.35
S8	162.42	352.36	351.77	−0.17	97.51	96.54	−0.99
S9	76.65	137.22	136.67	−0.40	79.35	80.11	0.96
S10	72.07	104.73	103.82	−0.87	135.57	134.61	−0.71
S11	154.23	309.27	308.73	−0.17	102.24	101.81	−0.42
S12	63.67	98.22	98.12	−0.10	116.00	115.63	−0.32
S13	63.02	97.46	97.08	−0.39	114.26	114.95	0.60
S14	82.52	234.76	234.73	−0.01	261.21	261.09	−0.05
S15	66.13	104.19	104.18	−0.01	111.98	112.00	0.02
S16	68.12	98.34	98.79	0.46	69.06	69.84	1.13
S17	78.46	250.43	250.31	−0.05	70.26	70.39	0.19
S18	63.51	99.26	99.17	−0.09	79.52	79.87	0.44

**Table 5:** HPLC elution conditions.

Time (min)	A (%)	B (%)
0	5	95
5	10	90
10	15	85
13	18	82
25	21	79
40	25.5	74.5
43	26.5	73.5
45	27	73
52	27.5	72.5
55	28	72
58	28	72
60	29	71
63	29	71
70	30	70
78	75	25
85	85	15
90	90	10
110	90	10

## Data Availability

The data are contained within the article.
